# Dietary Goji Shapes the Gut Microbiota to Prevent the Liver Injury Induced by Acute Alcohol Intake

**DOI:** 10.3389/fnut.2022.929776

**Published:** 2022-07-08

**Authors:** Lin Guo, Qijie Guan, Wenhui Duan, Yilin Ren, Xiao-Juan Zhang, Hong-Yu Xu, Jin-Song Shi, Fang-Zhou Wang, Ran Lu, Hui-Ling Zhang, Zheng-Hong Xu, Huazhong Li, Yan Geng

**Affiliations:** ^1^The Key Laboratory of Industrial Biotechnology, Ministry of Education, School of Biotechnology, Jiangnan University, Wuxi, China; ^2^National Engineering Research Center for Cereal Fermentation and Food Biomanufacturing, Jiangnan University, Wuxi, China; ^3^Jiangsu Provincial Engineering Research Center for Bioactive Product Processing, Jiangnan University, Wuxi, China; ^4^Key Laboratory of Carbohydrate Chemistry and Biotechnology, Ministry of Education, School of Life Sciences and Health Engineering, Jiangnan University, Wuxi, China; ^5^Department of Gastroenterology, Affiliated Hospital of Jiangnan University, Wuxi, China; ^6^Ningxia Red Power Goji Co., Ltd, Zhongwei, China; ^7^Ningxia Key Laboratory for Food Microbial-Applications Technology and Safety Control, Ningxia University, Yinchuan, China

**Keywords:** *Lycium barbarum* L., acute liver injury, metabolomics, gut microbiota, fecal microbiota transplantation (FMT)

## Abstract

Diet is a major driver of the structure and function of the gut microbiota, which influences the host physiology. Alcohol abuse can induce liver disease and gut microbiota dysbiosis. Here, we aim to elucidate whether the well-known traditional health food Goji berry targets gut microbiota to prevent liver injury induced by acute alcohol intake. The results showed that Goji supplementation for 14 days alleviated acute liver injury as indicated by lowering serum aspartate aminotransferase, alanine aminotransferase, pro-inflammatory cytokines, as well as lipopolysaccharide content in the liver tissue. Goji maintained the integrity of the epithelial barrier and increased the levels of butyric acid in cecum contents. Furthermore, we established the causal relationship between gut microbiota and liver protection effects of Goji with the help of antibiotics treatment and fecal microbiota transplantation (FMT) experiments. Both Goji and FMT-Goji increased glutathione (GSH) in the liver and selectively enriched the butyric acid-producing gut bacterium *Akkermansia* and Ruminococcaceae by using 16S rRNA gene sequencing. Metabolomics analysis of cecum samples revealed that Goji and its trained microbiota could regulate retinoyl β-glucuronide, vanillic acid, and increase the level of glutamate and pyroglutamic acid, which are involved in GSH metabolism. Our study highlights the communication among Goji, gut microbiota, and liver homeostasis.

## Introduction

The liver and gut have a bidirectional relationship in function and metabolites, which is called the gut–liver axis (1). The comorbid phenomenon of liver and intestinal diseases also suggested that there may be an interrelated pathogenesis. Heavy acute or chronic long-term drinking of alcohol can lead to acute liver injury (ALI) and alcoholic liver disease (ALD), which ranges from asymptomatic steatosis, alcoholic hepatitis, and cirrhosis (2, 3). Alcohol and its metabolites, such as acetaldehyde, are toxic to various types of liver cells, which can induce reactive oxygen species (ROS) (4), upregulate the inflammatory cascade (5), and lead to the damage of DNA or lipid peroxidation (6). Moreover, alcohol can also disrupt the gut–liver axis, including the mucus barrier, epithelial barrier (7), the production of antimicrobial peptide (8), and induce the gut dysbiosis (9), resulting in the elevation of pathogen-associated molecular patterns (PAMPs), such as lipopolysaccharide (LPS) and bacterial DNA (10). The resultant recognition and activation of Toll-like receptors and NOD-like receptors on liver immune cells contribute to the pathogenesis of alcoholic liver injury (11).

Clinical and pre-clinical studies have demonstrated that alcohol consumption can cause dysbiosis in the gut microbiota (9, 12). On the basis of the current understanding, “gut–liver axis” homeostasis is a major factor for alcoholic liver injury occurrence and progression. Modulation of gut microbiota could potentially prevent or attenuate liver injury by protecting gut barrier function and regulating gut microbiota metabolites, such as short-chain fatty acids (SCFAs) (13). Recently, more studies have proved that dietary intervention is considered an effective and healthy way to shape gut microbiota composition and influence host metabolism (14, 15). The dietary intervention is also regarded as a reliable strategy for preventing the occurrence and progression of alcoholic liver injury (16).

Goji (*Lycium barbarum* L., Goji berry, wolfberry) is a well-known traditional Chinese medicinal plant recorded in the Chinese Pharmacopeia, which also has long been consumed as a nourishing food or “Superfoods” in China, Europe, and around the world. Dietary Goji berry and its bioactive compounds are known to have beneficial effects on liver health, likely as a result of antioxidant and anti-inflammatory effects, as well as prebiotic properties (17–19). However, the effects of dietary Goji berry on the liver, gut microbiome, metabolites, and their interactions are still very limited.

Here, we assessed the effect of dietary intake of Goji in mice on acute ethanol challenge. We used the strategy of antibiotics treatment (ABX) and fecal microbiota transplantation (FMT) to elucidate the role of gut microbiota in the hepatoprotection of Goji. The Verrucomicrobia *Akkermansia* was highlighted to be significantly upregulated by Goji and positively correlated with the effect of Goji. In addition, the effect of Goji on cecum metabolism was investigated by metabolomics. The findings presented here support the hypothesis that the modulation of the gut microbiota is involved in the hepatoprotection effect of Goji, helping to elucidate the communication between prebiotic food, gut microbiota, and liver homeostasis.

## Materials and Methods

### Materials

Goji berry (*Lycium barbarum* L.) was supplied from the Ningxia Red Power Goji Co., Ltd (Ningxia, China). TRIzol reagent was purchased from Invitrogen (Carlsbad, CA, USA). Detection kits for ALT, AST, and GSH were provided by the Jiancheng Corp (Nanjing, China). Antibiotics and SCFAs reagents were purchased from Aladdin (Shanghai, China). EtOH and all other chemicals and materials were purchased from the Sangon Biotech (Shanghai, China).

### Ethical Statement

All experiments involving animals were conducted according to the ethical policies and procedures approved by the Committee of Ethics in Jiangnan University, China (Approval no. JN. No20181015c0321115[213] and JN. No20210430c1000713[109]). All of the procedures involving the use and care of animals complied with the guidelines of the European Community (Directive 2010/63/EU).

### Animals and Experimental Design

4-week-old male or female C57BL/6 mice were purchased from the Shanghai SLAC Laboratories Animal Co. Ltd (Shanghai, China). All the mice were housed in a specific-pathogen-free (SPF) environment with controlled conditions, a 12 h light/dark cycle at 20–22°C, and 45 ± 5% with *ad libitum* access to chow diet (AIN93-G) and water.

**Figure 1:**
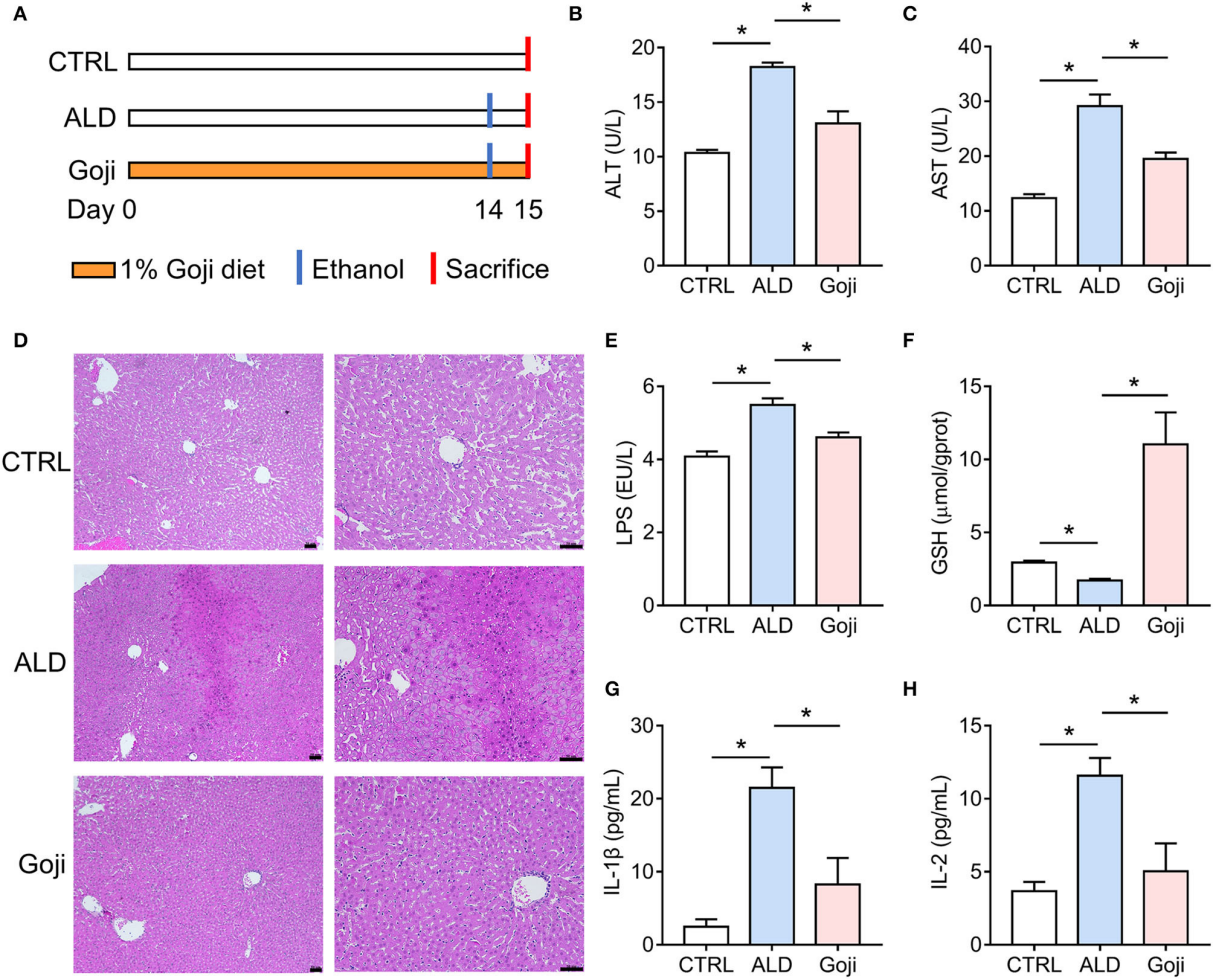
Dietary Goji prevented acute alcohol-induced liver injury in mice. **(A)** Experiment design. **(B)** AST level in serum. **(C)** ALT level in serum. **(D)** the representative sections of liver stained with hematoxylin and eosin (100× , 200× ). Scale bars, 50 μm. **(E)** The concentration of LPS in the liver. **(F)** the concentration of GSH in the liver. **(G)** the level of IL-1β in serum. **(H)** the level of IL-2 in serum. *n* = 6–8 for B–F; *n* = 3–4 for G and H. Data are shown as mean ± SEM, and statistical significance is assessed by one-way ANOVA corrected for multiple comparison by Tukey's test. *p-value < 0.05.

In the preventive treatment study, all mice were acclimated by placing them on a control chow diet administered *ad libitum* for 7 days, and then they were randomly divided into three groups (*n* = 16 per group). The control group (CTRL) and EtOH group (ALD) received a normal chow diet (AIN93-G), and the Goji group (Goji) received the same diet supplemented with 1% Goji (1% of dry feed weight) for 14 days (Figure 1A). Supplementary Table S1 shows the composition of diets (20). The human equivalent intake is approximately 13–20 g for an adult with a body weight of 60 kg per day.

In the antibiotic treatment study, mice were divided into three groups as ABX-CTRL, ABX-ALD, and ABX-Goji. All mice were given autoclaved drinking water for 14 days. Throughout the experiment, antibiotics were administered in sterile drinking water *ad libitum* and changed every 2 days (21).

In the FMT study, mice were pretreated with antibiotics as previously described (*n* = 8 per group) (22). Subsequently, the FMT study was performed as previously described, with some modifications (Figure 4E) (22). The fecal samples were freshly collected from the donor mice fed control or Goji diet for 14 days, and 3–5 fresh feces pellets (80–100 mg) were resuspended in 1 ml sterile PBS. After resuspension, tubes were centrifuged at 500 ×*g* for 1 min to remove insolubilized material, and mice were gavaged 200 ul supernatant (23).

To induce acute alcoholic liver injury, mice were gavaged a single shot of alcohol with 50% (vol/vol) EtOH at a dose of 12 ml/kg body weight, except for the CTRL, ABX-CTRL, and FMT-CTRL group. The control group mice received water by intragastric gavage.

At the end of the experiment, all mice were sacrificed at 12 h after the alcohol challenge with isoflurane. The blood was centrifugated at 3,000 ×g for 15 min to obtain the serum, which was then stored at −20°C for further analysis. After the liver and colon were removed, they were collected and stored in liquid nitrogen until further analysis or were stored in 4% paraformaldehyde for histological evaluation.

### Biochemical Analysis

The AST, ALT, and GSH levels in serum and liver were measured by commercial assay kits (Jiancheng, China) according to the manufacturer's instruction. LPS was measured by the ELISA kit (Enzyme-linked Biotechnology Co., Ltd., Shanghai). Cytokines and chemokines in serum were measured using a Bio-Plex Pro Mouse Cytokine Grp I Panel 23-Plex (No. #M60009RDPD, Bio-Rad, Austin, TX, USA) detection kit. Detection was completed by Wayen Biotechnologies (Shanghai) Inc. on a microbead suspension platform (Bio-Plex MAGPIX System (Bio-Rad)) (24).

### Histopathological Analysis

Parts of liver tissues were fixed in paraformaldehyde (4% in PBS) for 24 h, embedded in paraffin, and then serially sectioned at 5 μm. Standard hematoxylin and eosin staining according to the conventional method was performed on these sections. Nikon ECLIPSE TS100 (Tokyo, Japan) was used to capture images.

For alcian blue periodic acid-Schiff (AB-PAS) and MUC2 staining (MUC2 Polyclonal Antibody was purchased from Abcam, ab97386), a portion of the colon was processed for paraffin embedding, sectioned, and mounted on slides for histologic analysis as previously described (25).

## RNA Extraction and Quantitative Real-time Reverse-transcription PCR Analysis (qRT-PCR)

Total RNA was isolated from 50 mg of liver and colon tissues using the Trizol reagent (Thermo Fisher Scientific, MA. USA). Equal amounts of 500 ng RNA were used to synthesize cDNA. cDNA was used for qRT-PCR with the SYBR Green PCR Master Mix according to the manufacturer's protocol (Thermo Fisher Scientific, MA. USA). Gene expression of *Claudin1* was assessed by qRT-PCR and normalized to *Gapdh* expression level (26). All samples were run in duplicate in a single 96-well-reaction plate, and data were analyzed using the 2^−Δ*ΔCt*^ method. Primer sequences for qRT-PCR are shown in Supplementary Table S2.

### Short-Chain Fatty Acids Analysis

Cecum contents samples were prepared as previously described with modifications (27). Samples were weighed and recorded in milligrams. Add 10 times the volume of ultrapure water of the sample weight, and centrifuged at 4°C for 10 min (4,500 ×g). The supernatant fraction was collected and passed through a 0.22 μm nylon organic filter. An HPLC equipped with UV 210 nm and Waters XBridge^TM^ C_18_ column (4.6 × 250 mm, 5 μm) heated to 35°C was used to measure formic, acetic, propionic, butyric, and isovaleric acid in the sample filtrate. The mobile phase was acetonitrile–water (30:70, pH was adjusted to 3.0 with 0.5% phosphoric acid) at a flow rate of 0.7 mL/min, and the injection volume was 10 μl. The level of SCFAs was determined by the external standard calibration method.

### 16S rRNA Gene Sequencing Analysis

Genomic DNA of mice fecal microbiota was extracted using the QIAamp PowerFecal DNA Kit from Qiagen (Hilden, Germany) and then the 16S rRNA data were analyzed with QIIME v1.9.1 (28). The details about amplicon preparation, sequencing, and analysis are described in the Supplementary Material. The α-diversity of Shannon and Simpson and β-diversity of PCoA were calculated by the QIIME software. LDA coupled with effect size measurement (LefSe) was used to specifically identify significance among microbial communities at multiple biological levels on a Galaxy web platform (29).

### Untargeted Metabolomics Analysis

LC-MS was conducted at BioNovoGene Co., Ltd. (Suzhou, China). In brief, cecum content was individually collected, extracted, and subjected to LC-MS assay (vanquish, Thermo, and QE Focus, Thermo). The detailed information on metabolites detection is described in the Supplementary Material. Discriminating differential metabolites between two groups of samples were identified using a statistically significant threshold of Variable Importance in Projection (VIP) value (VIP ≥1) based on orthogonal partial least-squares discriminant analysis (OPLS-DA), and further validated by Student's *t*-test analysis (*p*-value ≤ 0.05).

### Statistical Analysis and Software

All data were presented as the mean ± SEM (standard error of the mean). Differences between the two groups were assessed using an unpaired two-tailed Student's *t*-test. Data sets involving more than two groups were assessed by one-way analysis of variance (ANOVA) corrected for multiple comparisons by Tukey's test or by the non-parametric Kruskal-Wallis test with Dunn's multiple comparisons test. A *p*-value < 0.05 was considered to be statistically significant.

Statistical analysis in this study was performed using the GraphPad Prism software (version 7.0, GraphPad Software, San Diego, California, USA). All plots were generated using the 4.0.3 R packages.

## Results

### Goji Supplementation Ameliorated Alcohol-Induced Liver Injury

To assess the protective effect of Goji on liver injury induced by acute alcohol intake, we did 14 days of intervention with 1% and 2% Goji diet, and positive control drug bifendatatum. We found that 1% and 2% Goji diet and bifendatatum have a hepatoprotective effect, as indicated by lower ALT and AST levels in serum (Supplementary Figure S1). Based on the proper daily consumption of Goji for humans, we chose a 1% Goji diet to further study the mechanisms of liver protection of Goji. We found that dietary Goji intervention for 14 days significantly lower serum ALT and AST levels (*p*-value < 0.05) (Figures 1A–C). And there was increased inflammatory cell infiltration on hematoxylin-eosin-stained liver sections in mice treated with ethanol, while this was attenuated by Goji supplementation (Figure 1D). LPS content was increased in the liver of ALD mice compared with CTRL mice. But the level of LPS was lower to 4.607 ± 0.1326 EU/L in Goji mice compared with the ALD mice (*p*-value < 0.05) (Figure 1E). Besides, there was a decreased level of glutathione (GSH) observed in the liver of ALD mice compared with CTRL mice, but the level markedly increased in the Goji pretreated mice (*p*-value < 0.05) (Figure 1F). Moreover, Goji significantly reduced the level of pro-inflammatory cytokines, such as IL-1β and IL-2, which were elevated by acute alcohol treatment (*p*-value < 0.05) (Figures 1G,H). Other inflammatory cytokines and chemokines, such as IL-3, IL-12, TNF-α, IL-17A, and MIP-1α, in the Goji group were significantly lower than in the ALD group (Supplementary Figure S2). The level of IL-4, IL-13, CCL2 (MCP-1), and CCL4 (MIP-1β) were also elevated in the serum of ALD mice, and Goji intervention showed a downward trend (Supplementary Figure S2). These results suggested that dietary Goji supplementation improved acute alcohol-induced inflammation and oxidative stress in the liver.

### Goji Maintained Epithelial Integrity and Modulated Short-Chain Fatty Acids

**Figure 2:**
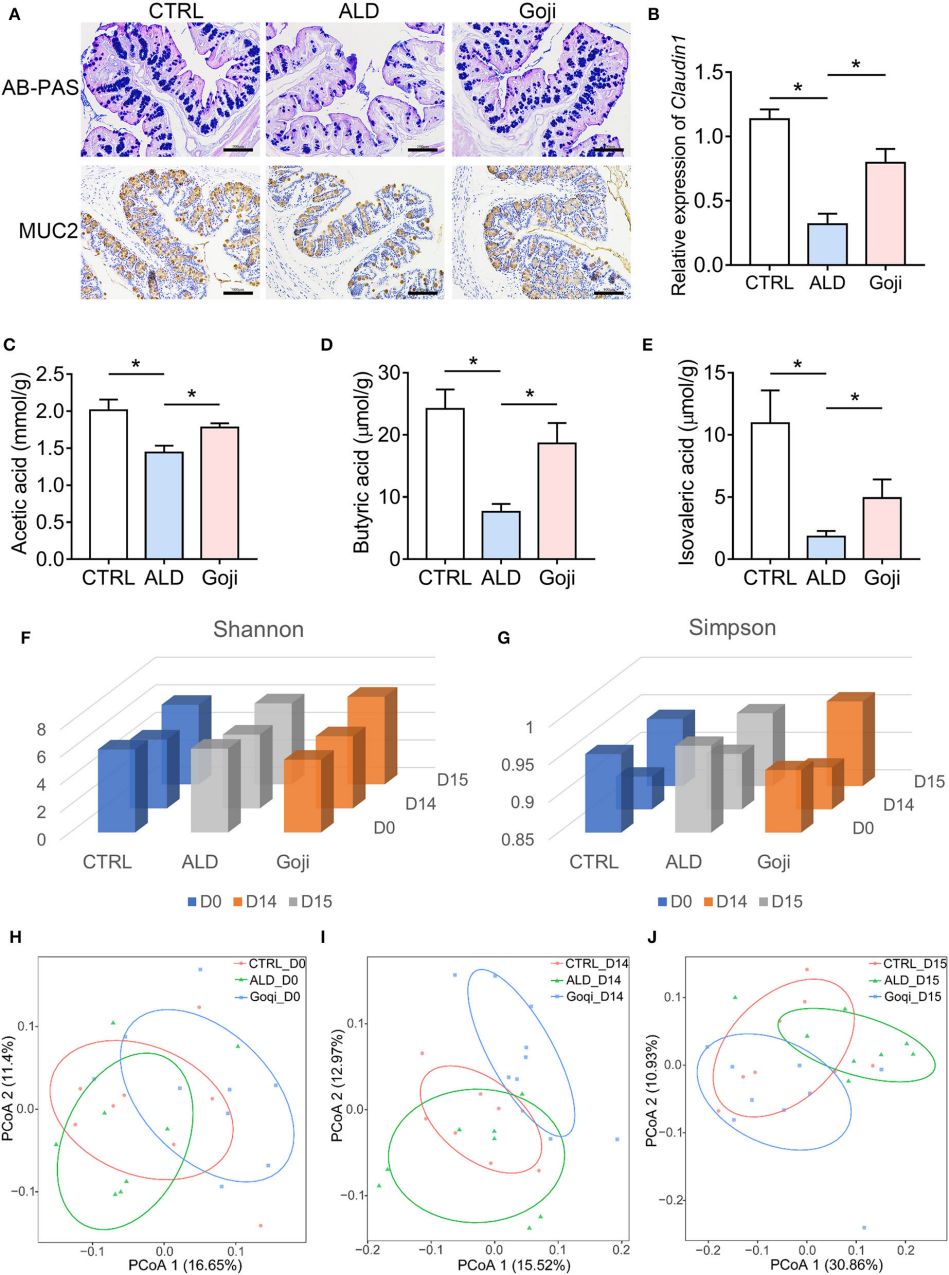
Goji maintained the gut microenvironment and modulated the overall structure of gut microbiota. **(A)** AB–PAS staining and immunohistochemical analysis of the expression of MUC2 of colon section. Scale bars, 100 μm. **(B)** The relative expression of Claudin1 in the colon. **(C)** quantification of cecal acetic acid. **(D)** quantification of butyric acid. **(E)** quantification of isovaleric acid. **(F)** Shannon index of microbiota; **(G)** Simpson index of microbiota. **(H)** principal-coordinate analysis (PCoA) plot at D0 based on unweighted UniFrac distance. Each point represents each sample. **(I)** PCoA plot at D14. **(J)** PCoA plot at D15. F–J, *n* = 8. Data are shown as mean ± SEM, and statistical significance is assessed by one-way ANOVA corrected for multiple comparison by Tukey's test. *p-value < 0.05.

Alcohol consumption could disrupt mucus and epithelial barrier (30) and trigger inflammatory reactions through the translocation of gut-derived PAMPs, such as LPS, an endotoxin derived from the outer membrane of Gram-negative bacteria (2). Because Goji inhibited LPS translocation to the liver, we next examined the effects of Goji on the intestinal barrier. Mucus is the first barrier of the intestine, and the bacteria or pathogens must penetrate it to reach the epithelial cells (31). The colonic surface goblet cells continuously secrete mucin2 (MUC2) and maintain the mucus layers. As shown in Figure 2A, the colon tissues of ALD mice exhibited a reduction in MUC2 and goblet cell number compared with that of CTRL mice, while Goji consolidated the mucus layer, as determined by MUC2 immunohistochemistry and AB-PAS staining. Alcohol decreased the gene expression of *Claudin1*, which is involved in the maintenance of intestinal integrity, while Goji treatment significantly elevated its expression in the colon tissues of mice (Figure 2B).

SCFAs are the most abundant metabolites derived from the fermentation of indigestible fiber-rich diets by colonic bacteria (13). We found that alcohol caused lower levels of SCFAs in the cecum contents compared with CTRL mice (Figures 2C–E). Goji supplementation induced an increase in acetic acid (Figure 2C) and nearly a 2-fold increase in butyrate compared to the ALD group (18.63 ± 3.296 and 7.621 ± 1.262 μmol/g, respectively, *p*-value < 0.05; Figure 2D). Besides, Goji also increased the level of isovaleric acid compared to ALD (4.919 ± 1.502, *n* = 7 and 1.817 ± 0.4527 μmol/g, *n* = 6, respectively, *p*-value < 0.05; Figure 2E). These data indicated that Goji restored the intestinal epithelial cell integrity and maintained the level of SCFAs in the gut.

### Goji Changed the Gut Microbial Composition

The commensal microbiota has a beneficial role in the protection against acute ALD (32). Therefore, we performed 16S rRNA gene sequencing to describe the overall changes in gut microbiota (33). Shannon and Simpson index exhibited no significant difference in α-diversity among the mice on days 0 (D0) and 14 (D14). After alcohol administration, the Shannon and Simpson indices were slightly higher in the Goji group than in the CTRL and ALD group (Figures 2F,G). Principal coordinates analysis (PCoA) of β-diversity based on an unweighted UniFrac distance reflected that Goji changed the gut microbiota structure in mice, especially after 14 days of feeding with Goji (Figures 2H–J).

Further comparison at D15 indicated that compared to the CTRL group, the ALD group had a higher relative abundance of Bacteroidetes and Proteobacteria but a lower relative abundance of Firmicutes and Epsilonbacteraeota, and Actinobacteria. Notably, Goji significantly reversed the phylum of Firmicutes, Bacteroidetes, and Proteobacteria changes (Supplementary Figure S3A). Besides, the relative abundance of Verrucomicrobia in the Goji group was significantly higher than in the CTRL and ALD groups. At the family level, Goji supplementation resulted in an increased abundance of Akkmansiaceae and Bifidobacteriaceae, but decreased the abundance of Prevotellaceae (Supplementary Figure S3B). At the genus level, Goji significantly increased the relative abundance of *Akkermansia* and *Ruminococcaceae_UCG_014*, while decreasing the relative abundance of *Alloprevotella* (Supplementary Figure S3C).

**Figure 3:**
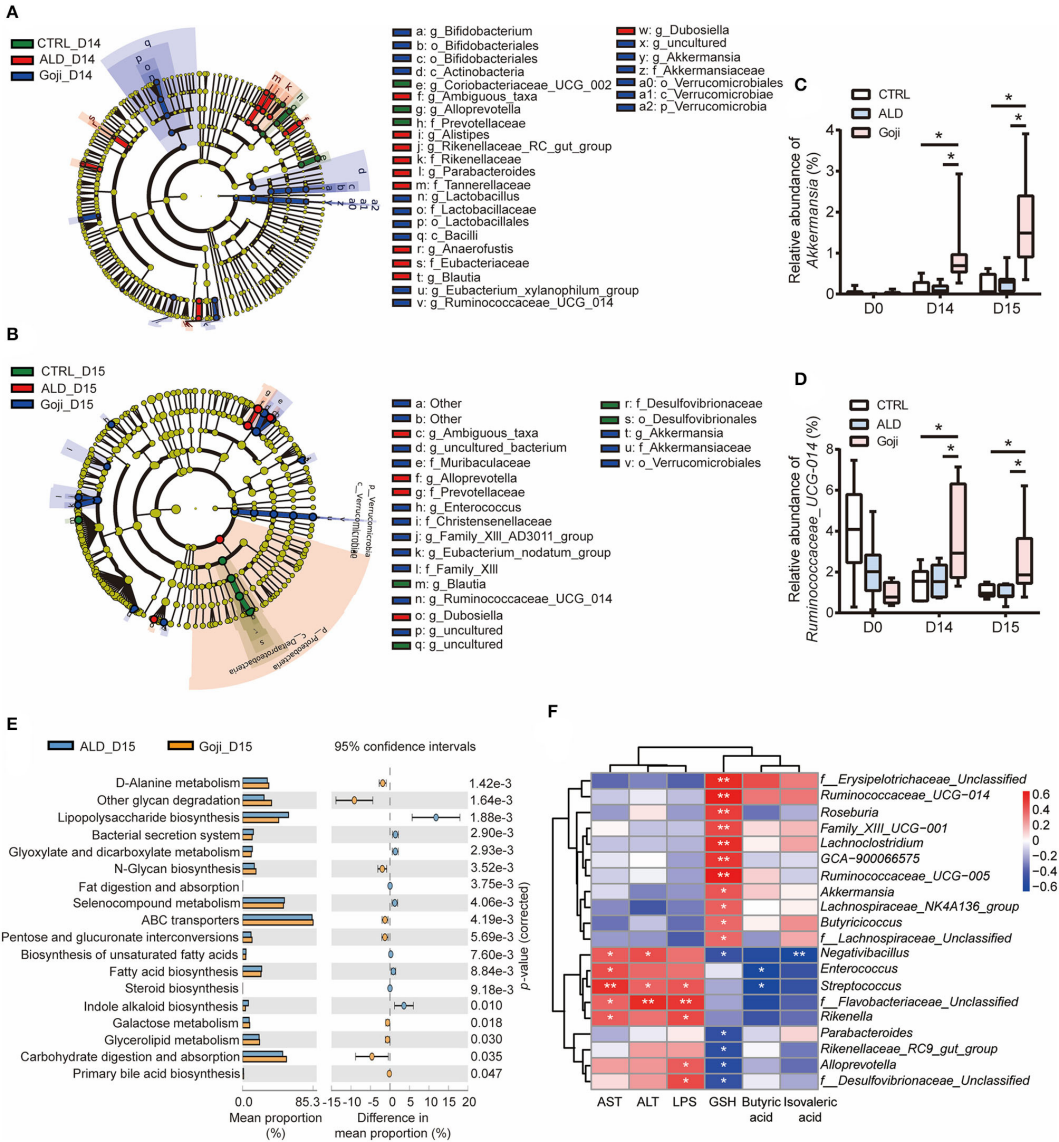
Significantly different taxa between experimental groups and prediction of metabolic function. **(A)** LEfSe taxonomic cladograms are processed to identify specific taxa at different levels after Goji pretreatment for 14 days (D14). **(B)** specific taxa between CTRL, ALD, and Goji groups after alcohol shock at D15. Significantly discriminant taxon nodes are colored, and branch areas are shaded according to the effect size of taxa. Green taxa enriched in CTRL group; Red taxa enriched in ALD group; Blue taxa enriched in Goji group. Only the taxon whose LDA score >3 is displayed. The species with no significant difference were colored yellow. Prefixes represent abbreviations for a taxonomic rank of each taxon, with phylum (p), class (c), order (o), family (f), and genus (g). **(C)** relative abundance of *Akkermansia*. **(D)** relative abundance of *Ruminococcaceae_UCG_014*. Data are shown as mean ± SEM (*n* = 7–8), and statistical significance assessed by one-way ANOVA corrected for multiple comparison by Tukey's test. **(E)** significant differences in metabolism pathway between ALD and Goji at Day 15. *P*-values were calculated using Welch's test. **(F)** the Spearman's correlation between the liver-related index and the relative abundance of significantly different genera. The scale represents the correlation coefficients. *p-value < 0.05, **p-value < 0.01.

To identify the differentially abundant taxonomy of the three experimental groups before and after alcohol exposure, we applied linear discriminant analysis (LDA) and LEfSe analysis (Figures 3A,B; Supplementary Figures S4, S5). LEfSe emphasizes the statistical significance and biological relevance. Based on LDA score, *Bifidobacterium, Lactobacillus, Eubacterium_xylanophilum_group, Ruminococcaceae _UCG_014*, and *Akkermansia* were the most abundant genus in the Goji group at D14. After acute alcohol exposure, *Akkermansia* and *Ruminococcaceae_UCG_014* were still enriched in the Goji group.

Then, we assessed the longitudinal change of relative abundance of genus *Akkermansia* and *Ruminococcaceae_UCG_014* during the experiment period. We confirmed that the relative abundance of *Akkermansia* and *Ruminococcaceae_UCG_014* was significantly enriched due to Goji supplementation after 14 days. The relative abundance of both genera in the Goji group was almost two times higher than the CTRL group on day 14, and their relative abundance was even higher after the alcohol challenge (*p*-value < 0.05) (Figures 3C,D). These data suggested that Goji shaped the gut microbiota, especially by increasing the relative abundance of *Akkermansia* and *Ruminococcaceae_UCG_014*.

### Goji Changed the Gut Microbial Function

The gut microbiota assumes vital physiological functions in the host. Functional genes are important to confer the metabolic phenotypes of microbes, leading to complex ecological interaction (34). We further performed Tax4Fun analysis (35) to predict functional target profiles at D15. There was a reduction in LPS biosynthesis in the Goji group compared to the ALD group. Besides, Goji upregulated other glycan degradation, carbohydrate digestion and absorption, and N-glycan biosynthesis in the Kyoto Encyclopedia of Genes and Genomes (KEGG) pathway analysis (Figure 3E).

Furthermore, we correlated liver injury-related parameters with 20 differential genera (relative abundance >0.1%) between ALD and Goji group by Spearman rank correlation analysis (Figure 3F). Among them, *Akkermansia* and *Ruminococcaceae_UCG_014* were positively correlated with GSH content (*r* = 0.49, *p*-value < 0.05; *r* = 0.68, *p*-value < 0.01, respectively) and negatively correlated with AST (*r* = −0.03, *p*-value = 0. 9; *r* = −0.07, *p*-value = 0.73, respectively) and ALT (*r* = −0.24, *p*-value = 0.31; *r* = 0.005, *p*-value = 0.98, respectively). While *Alloprevotella* and *f_Desulfovibrionaceae_unclassified* were significantly positively correlated with LPS, but negatively correlated with GSH content (Figure 3F).

### Goji-Trained Gut Microbiota Prevented Acute Alcohol-Induced Liver Injury

**Figure 4:**
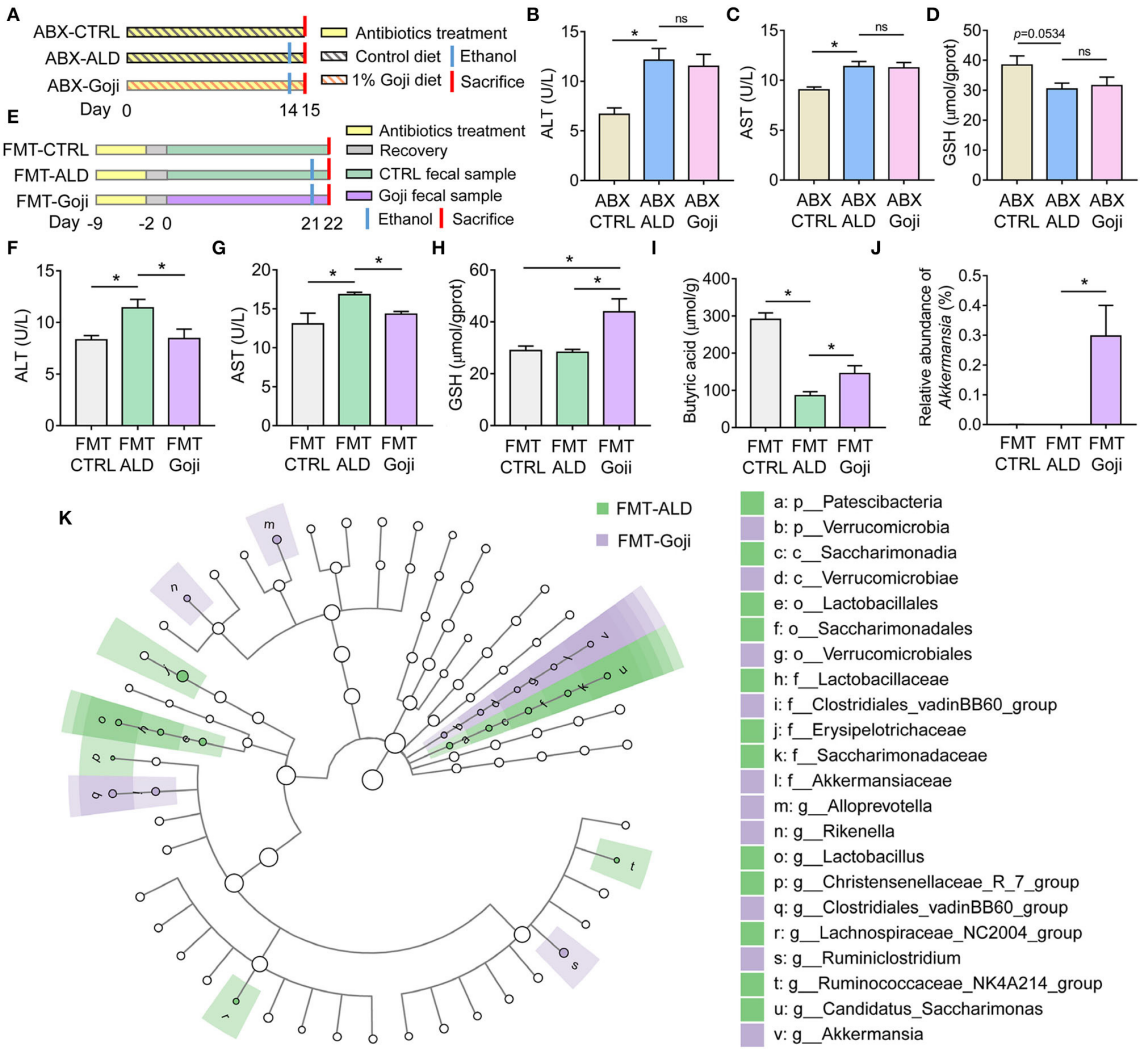
The liver protective effect of Goji is dependent on gut microbiota. **(A)** ABX treatment experiment design. **(B)** the content of ALT in serum. **(C)** the content of AST in serum. **(D)** the content of GSH in liver tissue. **(E)** experiment design. Fecal samples from the CTRL group were transplanted to the pseudo-germ-free mice by antibiotics pretreatment and divided into groups: FMT-CTRL and FMT-ALD. The FMT-Goji group transplanted the fecal samples from the Goji pretreated mice. Both FMT-ALD and FMT-Goji groups were gavaged alcohol to induce alcohol liver injury. **(F)** the content of AST in serum. **(G)** the content of AST in serum. **(H)** the content of GSH in liver tissue. **(I)** quantification of cecal butyric acid. **(J)** relative abundance of *Akkermansia*. **(K)** significantly different taxa between FMT-ALD and FMT-Goji group analyzed by LEfSe analysis. Data are shown as mean ± SEM, and statistical significance is assessed by one-way ANOVA corrected for multiple comparison by Tukey's test. *p-value < 0.05.

To determine whether gut microbiota may contribute to the hepatoprotection effect of Goji, we performed ABX treatment and FMT. As shown in Figure 4A, mice were treated with ABX during the whole experiment. We found the liver protective effect of Goji was abrogated with the elimination of gut microbes (Figures 4B–D).

To further examine whether the modulated gut microbiota structure by Goji may play a causal effect on liver protection, an FMT experiment was performed. We found mice transplanted with Goji-trained microbiota exhibited a significantly decreased level of ALT and AST in serum compared to mice transplanted with CTRL microbiota after alcohol treatment (*p*-value < 0.05) (Figures 4E–G). And the inflammation of the liver was alleviated in FMT-Goji (Supplementary Figure S6A). Notably, FMT-Goji significantly increased the GSH content in the liver compared with FMT-CTRL and FMT-ALD groups (*p*-value < 0.05) (Figure 4H). Compared with the FMT-ALD group, the FMT-Goji group led to a 1.69-fold increase of butyric acid in the cecum (*p*-value < 0.05) (Figure 4I). The level of acetic acid and propionic acid has no significant difference between FMT-ALD and FMT-Goji group (Supplementary Figures S6B,C). Moreover, the relative abundance of *Akkermansia* was higher in the FMT-Goji group compared with the FMT-CTRL and FMT-ALD groups (*p*-value < 0.05) (Figure 4J). The relative abundance of family Ruminococcaceae was higher in FMT-Goji than in FMT-CTRL and FMT-ALD groups (Supplementary Figure S6D). LEfSe analysis revealed that the genus of *Ruminiclostridium* and *Akkermansia* were the biomarker in FMT-Goji mice (Figure 4K). These data suggested that the liver protective ability of Goji could be transferred by fecal microbiota. Furthermore, the gut microbiota is required for Goji to increase endogenous GSH in the liver to protect its function.

### Goji and Goji-Trained Gut Microbiota Altered Metabolic Profiles

**Figure 5:**
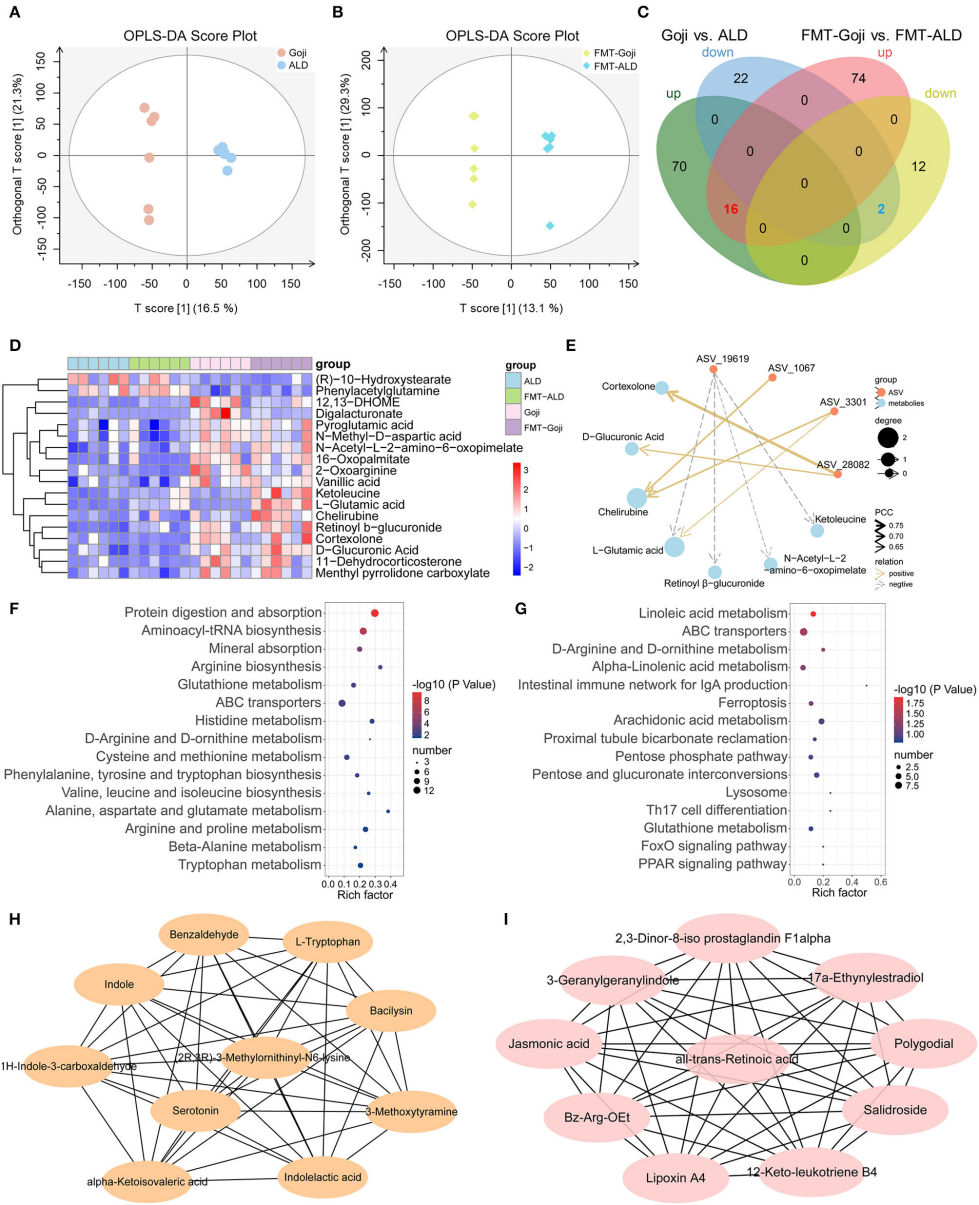
Metabolic patterns in Goji vs. ALD and FMT-Goji vs. FMT-ALD mice. **(A)** The orthogonal partial least-squares discriminant analysis (OPLS-DA) of Goji vs. ALD. **(B)** OPLS-DA of FMT-Goji vs. FMT-ALD. **(C)** Venn diagrams of differential metabolites between Goji vs. ALD, and FMT-Goji vs. FMT-ALD. **(D)** heatmap of 16 upregulated and 2 downregulated metabolites in Goji and FMT-Goji group compared with ALD and FMT-ALD groups. **(E)** correlation network of metabolites and ASV. **(F)** KEGG enrichment pathways based on the differential metabolites in Goji vs. ALD. **(G)** KEGG enrichment pathways based on the differential metabolites in FMT-Goji vs. FMT-ALD. **(H)** the 10 metabolites in hub-network of DEMs between Goji and ALD. **(I)** the 10 metabolites in hub-network of DEMs between Goji and ALD.

Bacterial metabolites influence the host in numerous ways. To investigate the effects of dietary Goji and Goji-trained gut microbiota on metabolite profiles, we examined metabolites of the cecal contents in a non-targeted manner. Using the OPLS-DA, the cecal content samples from distinct groups could be largely separated (Figures 5A,B). By using a threshold of *p*-value < 0.05 and VIP > 1, differentially expressed metabolites (DEMs) between Goji and ALD, and FMT-Goji and FMT-ALD were identified (Figure 5C). Based on the Venn analysis, 16 metabolites were upregulated, and two metabolites were downregulated in Goji and FMT-Goji compared with ALD and FMT-ALD, respectively (Supplementary Table S3; *p*-value <0.05). Heatmaps of the intensities of these 18 metabolites are shown in Figure 5D. Among these metabolites, (R)-10-hydroxystearate and phenylacetylglutamine were downregulated in Goji and FMT-Goji compared with ALD and FMT-ALD. And the levels of L-glutamic acid, pyroglutamic acid, retinoyl β-glucuronide, vanillic acid, chelirubine, D-glucuronic acid, 2-oxoarginine, ketoleucine, 12,13-DHOME, 16-oxopalmitate, N-acetyl-L-2-amino-6-oxopimelate, N-methyl-D-aspartic acid, and digalacturonate were notably reversed in Goji and FMT-Goji.

To explore the relationship between the co-regulation of gut microbiota and metabolites, Pearson correlation analysis was carried out and the correlation coefficient network was obtained (Figure 5E; Supplementary Table S4). ASV_3301 and ASV_1067 belonging to *Akkermansia* were positively related to chelirubine. ASV_3301 was also positively related to L-glutamic acid.

Moreover, the top 15 significantly differential KEGG pathways based on DEMs between Goji and ALD, and FMT-Goji and FMT-ALD are shown in Figures 5F,G. We found Goji and FMT-Goji both regulated GSH metabolism. We further calculated a network of hub metabolites based on maximal clique centrality (MCC). The main upregulated hub metabolites in Goji were (2R,3R)-3-methylornithinyl-N6-lysine indole, L-tryptophan, 1H-indole-3-carboxaldehyde, indoleacetic acid, and serotonin (Figure 5H). The main upregulated hub metabolites in FMT-Goji were retinoyl β-glucuronide, 2,3-dinor-8-iso prostaglandin F1alpha, 3-geranylgeranylindole, jasmonic acid, polygodial, lipoxin A4, and 12-keto-leukotriene B4 (Figure 5I). These data indicated that the metabolites produced by gut microbes and hosts may function as a mediator in the liver protection function of Goji.

## Discussion

Crosstalk between the gut and liver is recognized as an important determinant of alcohol-induced liver disease (1). Therefore, modulation of gut microbial communities and metabolites is needed, where the metabolites, such as SCFAs, could provide an energy substance for colonocytes and alleviate intestinal inflammation (13). Diet is a simple and effective method to rapidly shift the intestinal microenvironment (15). Moreover, given the role of the gut microbiota, FMT has been investigated in alcohol-related liver disease for numerous applications (36). Goji has been widely studied because of its multiple functional activities, and whether the protective effects are associated with gut microbiota has attracted more attention. Here, Goji was supplied in the diet, as a simulation of about 13–20 g/day for an adult. Our study found that Goji could modulate gut microbiota composition and enrich the butyrate-producing bacteria, such as *Akkermansia* and *Ruminococcaceae_UCG_014*. Goji supplementation also maintained the integrity of colonic mucus, and inhibited the intrude of LPS into liver tissue through the portal vein under alcohol administration. Subsequently, the elevated level of transaminase and pro-inflammatory cytokines induced by acute alcohol administration in serum was decreased. The level of antioxidant GSH was increased in the liver tissue after Goji supplementation. In addition, we used ABX and FMT to elucidate that gut microbiota is a critical factor for Goji to prevent liver injury induced by acute alcohol intake.

The 16S rRNA gene sequencing results revealed that there was a significant difference in the structure of gut microbiota between the Goji treated group and the untreated group after the once acute massive gavage of alcohol. Previous studies have confirmed that alcohol intake is positively correlated with an increase in endotoxin-producing bacteria and negatively correlated with a reduction in SCFAs-producing bacteria (37). Here, we found that Goji dietary intervention after 14 days significantly changed the community structure of gut microbiota in mice. The increased relative abundance of Enterobacteriaceae related to endotoxin-producing bacteria after alcohol administration was confirmed in our study (38). In our study, the abundance of phylum Firmicutes (39), which harbors many SCFAs-producing bacteria, and other SCFAs producers, such as family Lachnospiraceae, genus *Akkermansia, Ruminococcaceae_UCG_005* (40), *Ruminococcaceae_UCG_014* (41), *Ruminiclostridium_5* (42), and *Christensenellaceae_R-7_group* (43), were more abundant in Goji compared to ALD group. SCFAs are potentially suitable metabolic candidates that could account for the effects of Goji. A previous study has shown that patients with alcoholic steatohepatitis (ASH) exhibited a decreased abundance of fecal *A. muciniphila* compared to healthy controls that indirectly correlated with hepatic disease severity. Oral *A. muciniphila* could protect against alcohol-induced gut leakiness, enhanced mucus thickness, and tight-junction expression in mice model of ALD (44). Moreover, the *Akkermansia* was able to produce a pili-like protein that enhances immune homeostasis in the gut mucosa as well as gut barrier function. Recent researches have revealed that several substances of Goji are known to increase the abundance of *Akkermansia*, such as polysaccharide (45, 46), and 2-O-β-d-Glucopyranosyl-l-ascorbic acid (47).

Microbial metabolites, such as SCFAs, which have been reported to be changed by alcohol consumption (48). Polysaccharides of Goji have been proved to enrich the concentration of SCFAs (49). In this study, we found that prophylactic Goji both increase the level of isovaleric acid and butyric acid beyond alcohol administration. Importantly, transplantation with Goji-pretreated feces to the mice also defended the decrease of butyric acid under alcohol stimulation. Butyrate is considered to be essential for maintaining intestinal barrier integrity by modulating the expression of tight junction proteins and mucin (50, 51). Alcohol feeding can induce glycosylation alteration of mucus glycoproteins, and it may lead to unbalance between protective and adhesive functions of mucus and disruption of the intestinal barrier. AB-PAS and MUC2 staining results indicated that Goji could maintain the integrity of mucus.

For functional analysis, the metabolism of LPS biosynthesis was downregulated in the Goji group compared with the ALD group. The pathogenesis of alcoholic liver injury is complex, as it not only results from the direct toxic effects of alcohol and its metabolites on cells, but other factors also play an important role in its pathogenesis, such as impairing lipid metabolism, intensifying inflammatory reactions, and inducing fibrosis (52). Moreover, microbial products and metabolites also correlated to alcohol-related liver diseases, including bile acids, indole-3-acetic acid, butyrate, long-chain fatty acids, cytolysin, β-glucan, and endotoxin (53). The increase in LPS biosynthetic potential may be consistent with an increased representation of Gram-negative Proteobacteria in steatosis, as observed in rodents (54). In this study, we found phylum Proteobacteria was significantly lower in the Goji group than in the ALD and CTRL groups.

More importantly, we found that the feces transplant from Goji-administrated donor mice defended the alcohol-induced liver injury of recipient mice. Remarkably, the GSH level in the liver tissue was also higher in FMT-Goji mice compared with FMT-CTRL and FMT-ALD mice. GSH is a major endogenous antioxidant in hepatocytes, and blocking the GSH export was found to delay or suppress apoptosis (55). Combined with the ABX experiments, our study revealed that Goji promoted the synthesis of GSH in the liver, which depends on gut microbiota. Interestingly, the abundance of *Akkermansia* was also enriched in the FMT-Goji group. Based on these results, the hepatoprotective effects of Goji may through promoting the growth of *Akkermansia*.

The metabolic profiles of cecum content showed that (R)-10-hydroxystearate, the substrate for the formation of oleic acid, and phenylacetylglutamine, which was related to the liver damage (56), were both downregulated by Goji and Goji-trained microbiota. Goji and Goji-trained microbiota significantly increase the content of the digalacturonate of pectin metabolic product and vanillic acid associated with antioxidant (57) and anti-inflammatory (58). In the hub network of metabolites, we found that Goji regulated some metabolites in tryptophan metabolism, and significantly increased the level of indole and indoleacetic acid that act as Aryl hydrocarbon receptor (AhR) ligands. The activation of AhR usually reduces inflammation in mice and maintains gut homeostasis (59). In FMT-Goji, the key point contributing to the metabolite correlation network is retinoyl β-glucuronide. The retinoyl β-glucuronide, a retinoid that is retinoic acid, was upregulated by Goji and its trained microbiota. It is a naturally occurring, biologically active metabolite of vitamin A. Retinol is vital to maintaining the integrity of the epithelium and mucous membranes (60). Previous studies showed that symbiotic bacteria produced retinol derivatives that could protect the intestinal epithelial barrier (61, 62). Besides, Goji and its trained microbiota also upregulated the levels of L-glutamic acid and pyroglutamic acid, which are involved in GSH metabolism. Predicting the metabolic function of gut microbiota may provide us with a deeper understanding of co-metabolism between gut microbiota and host and clarify the mechanism of Goji further.

The animal experimental design has some limitations. Binge drinking is a risk factor for liver disease. Several animal models have been considered to examine the effects of binge alcohol administration, including single binge, intermittent repeat binge, and chronic alcohol exposure followed by episodes of binge (63, 64). Single alcohol administration has been used to study the mechanism of the acute pathogenesis of alcoholic liver injury (11, 65–70). The single binge episode by administering alcohol to mice represents the early-stage acute alcoholic liver injury, and the protective effects of Goji on different stages of alcohol-induced liver injury remain to be further studied. The other limitation of this study is that how gut microbes affect the endogenous liver GSH synthesis has not been fully explained. And how components/compounds of Goji precisely modulate gut microbiota need future investigation. We also look forward to using human trials to better study the role of Goji in shaping the microbial ecosystem.

## Conclusion

Our results revealed that Goji had a hepatoprotective effect indicated by alleviating inflammation and improved antioxidant capacity under binge alcohol intake. Goji also maintains the gut barrier in mice by shaping the gut microbial community, especially enhancing *Akkermansia*. Goji and its trained microbiota can markedly decrease toxic metabolites, such as phenylacetylglutamine, and significantly increase the colonic SCFAs, L-glutamic acid, pyroglutamic acid, vanillic acid, and retinoyl β-glucuronide. Moreover, the GSH modulated by Goji is relevant to the microbes.

Goji may restore the gut microbiota homeostasis by improving the gut microbiota dysfunction and metabolites. Additionally, this study indicated that the combined application of multi-omics might be a valuable strategy for discovering prebiotic food mechanisms.

## Data Availability Statement

The original data we used in this study can be downloaded with following links: https://www.ncbi.nlm.nih.gov/bioproject/?term=PRJNA809100.

## Ethics Statement

The animal study was reviewed and approved by the Institutional Animal Care and Use Committee of Jiangnan University, Wuxi, China.

## Author Contributions

LG, YG, H-LZ, HL, and Z-HX designed this study. LG, QG, WD, and YR performed experimental work and analyzed data. QG, X-JZ, H-YX, and J-SS examined and revised methodology. F-ZW and RL provided resources. LG wrote the first draft of the manuscript. YG, HL, and Z-HX supervised and interpreted the experimental data, and critically revised the manuscript. All authors contributed to the article and approved the submitted version.

## Funding

This work was supported by the grants from the National Natural Science Foundation of China (Grant Nos. 31970746 and 31771514), the Ningxia Hui Autonomous Region's Key Research and Development Plan (Grant No. 2020BBF02023), the Key Research and Development Program of Ningxia Hui Autonomous Region (Grant No. 2017BY069), the Science and Technology Innovation Team Foundation of Ningxia Hui Autonomous Region (KJT2017001), and the Qing Lan Project in Jiangsu Province.

## Conflict of Interest

F-ZW and RL are employed by the company Ningxia Red Power Goji Co., Ltd. The remaining authors declare that the research was conducted in the absence of any commercial or financial relationships that could be construed as a potential conflict of interest.

## Publisher's Note

All claims expressed in this article are solely those of the authors and do not necessarily represent those of their affiliated organizations, or those of the publisher, the editors and the reviewers. Any product that may be evaluated in this article, or claim that may be made by its manufacturer, is not guaranteed or endorsed by the publisher.

## Abbreviations

IL-1β: interleukin-1β
IL-2: interleukin-2
FMT: fecal microbiota transplantation
GSH: glutathione
ALD: alcohol liver disease
ROS: reactive oxygen species
PAMPs: pathogen-associated molecular patterns
LPS: lipopolysaccharide
SCFAs: short-chain fatty acids
ABX: antibiotics
PCA: principal component analysis
DEGs: differentially expressed genes
MUC2: mucin2
AB-PAS: alcian blue periodic acid-Schiff
PCoA: principal coordinates analysis
LDA: linear discriminant analysis
LEfSe: LDA effect size
DEMs: differentially expressed metabolites
ASH: alcoholic steatohepatitis
GO: gene ontology
KEGG: Kyoto Encyclopedia of Genes and Genomes
PPI: protein–protein interaction
MCODE: molecular complex detection
VIP: variable importance in projection.
