# Supplementary material for: Dietary Goji Shapes the Gut Microbiota to Prevent the Liver Injury Induced by Acute Alcohol Intake
Source: Front Nutr. 2022 Jul 8;9:929776. doi: 10.3389/fnut.2022.929776 (PMC9309278; doi:10.3389/fnut.2022.929776)
Supplement: Supplementary file 1 [file Data_Sheet_1.DOCX]

Supplementary Material

# RNA Sequencing Data Analysis

The raw reads of RNA sequencing were cleaned by removing adaptors and low-quality reads using SOAPnuke (1). After filtering, HISAT software was used to map the clean reads to the *Mus musculus* (GRCm38.p6) reference genome (GCF_000001635.26), with approximately 93.49% of reads mapping uniquely. We used Bowtie2 (v2.2.5) (2) to align clean reads to the reference gene sequence and then used RSEM (3) to calculate the gene expression values of each sample. The read counts were used for differential gene expression analysis using DESeq2 (4).

# 16S rRNA Gene Sequencing Data Analysis

The DNA of microbiota was used for amplification of V3-V4 hypervariable regions of the 16S rRNA gene the forward primer (5’-CCT ACG GRR BGC ASC AGK VRV GAA T-3’), and the reverse primer (5’-GGA CTA CNV GGG TWT CTA ATC C-3’). (1) The two sequences were aligned and spliced according to the end overlapping region of the alignment. At least 20 bp overlapping region was ensured during splicing, and the sequence containing N was removed from the splicing result;(2) The primer and the joint sequence were removed, the base group with the mass value of both ends lower than 20 was removed, and the sequence with the length less than 200 bp was discarded; (3) The above spliced and filtered sequences were compared with the database, and the chimera sequence was removed to get the final valid data. DNA libraries were multiplexed and loaded on an Illumina MiSeq instrument according to the manufacturer’s instructions (Illumina, San Diego, CA, USA). The effective sequences were used in the final analysis. Sequences clustered into operational taxonomic units (OTUs) at a 97% identity threshold using an open-reference OTU picking approach with VSEARCH (1.9.6) (5) against the Silva_132 16S rRNA database (http://www.arb-silva.de/), and then perform taxonomic analysis on the representative sequence of OTU (6).

# LC-MS Analysis

Chromatographic separation was used with an ACQUITY UPLC® HSS T3 (150×2.1 mm, 1.8 μm, Waters) column maintained at 40 ℃. The temperature of the autosampler was 8 ℃. Gradient elution of analytes was carried out with 0.1% formic acid in water (C) and 0.1% formic acid in acetonitrile (D) or 5 mM ammonium formate in water (A) and acetonitrile (B) at a flow rate of 0.25 mL/min. Injection of 2 μL of each sample was done after equilibration. An increasing linear gradient of solvent B (v/v) was used as follows: 0~1 min, 2% B/D; 1~9 min, 2%~50% B/D; 9~12 min, 50%~98% B/D; 12~13.5 min, 98% B/D; 13.5~14 min, 98%~2% B/D; 14~20 min, 2% positive model (14~17 min, 2% B-negative model). The ESI-MSn experiments were used with the spray voltage of 3.5 kV and -2.5 kV in positive and negative modes, respectively. Sheath gas and auxiliary gas were set at 30 and 10 arbitrary units, respectively. The capillary temperature was 325 ℃. respectively. The Orbitrap analyzer scanned over a mass range of m/z 81-1 000 for full scan at a mass resolution of 70 000. Data dependent acquisition (DDA) MS/MS experiments were performed with HCD scan. The normalized collision energy was 30 eV. Dynamic exclusion was implemented to remove some unnecessary information in MS/MS spectra. The metabolite annotation of the LC-MS data was performed with the Compound Discoverer program and referenced to the mzCloud database (www.mzCloud.org), as well as the Human Metabolome Database (www.hmdb.ca), METLIN (metlin.scripps.edu), MassBank (www.massbank.jp), and LIPID MAPS (www.lipidmaps.org).

# Supplementary Figures

**
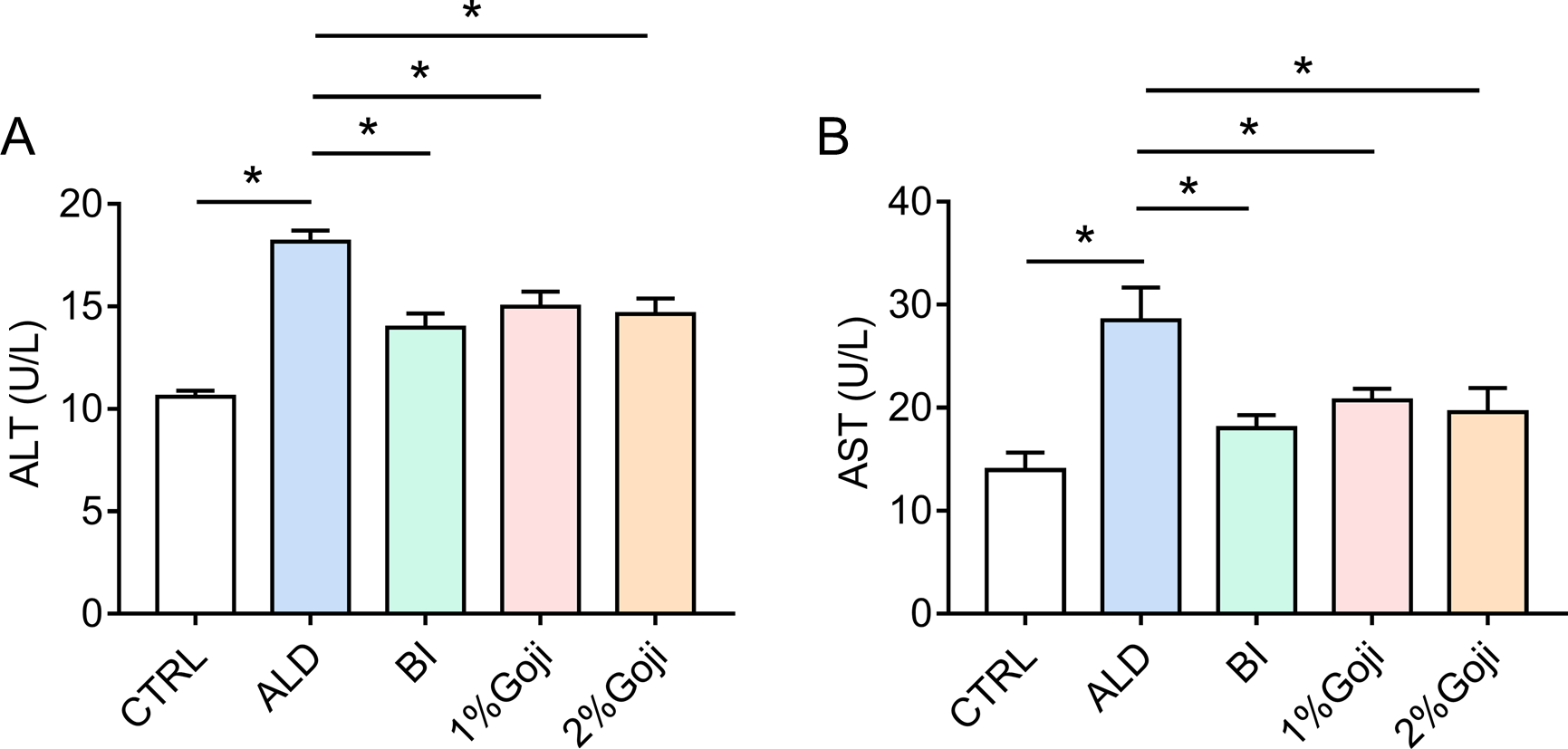
**

**Supplementary Figure 1. (A)** The level of ALT; **(B)** The level of AST in the serum.

**
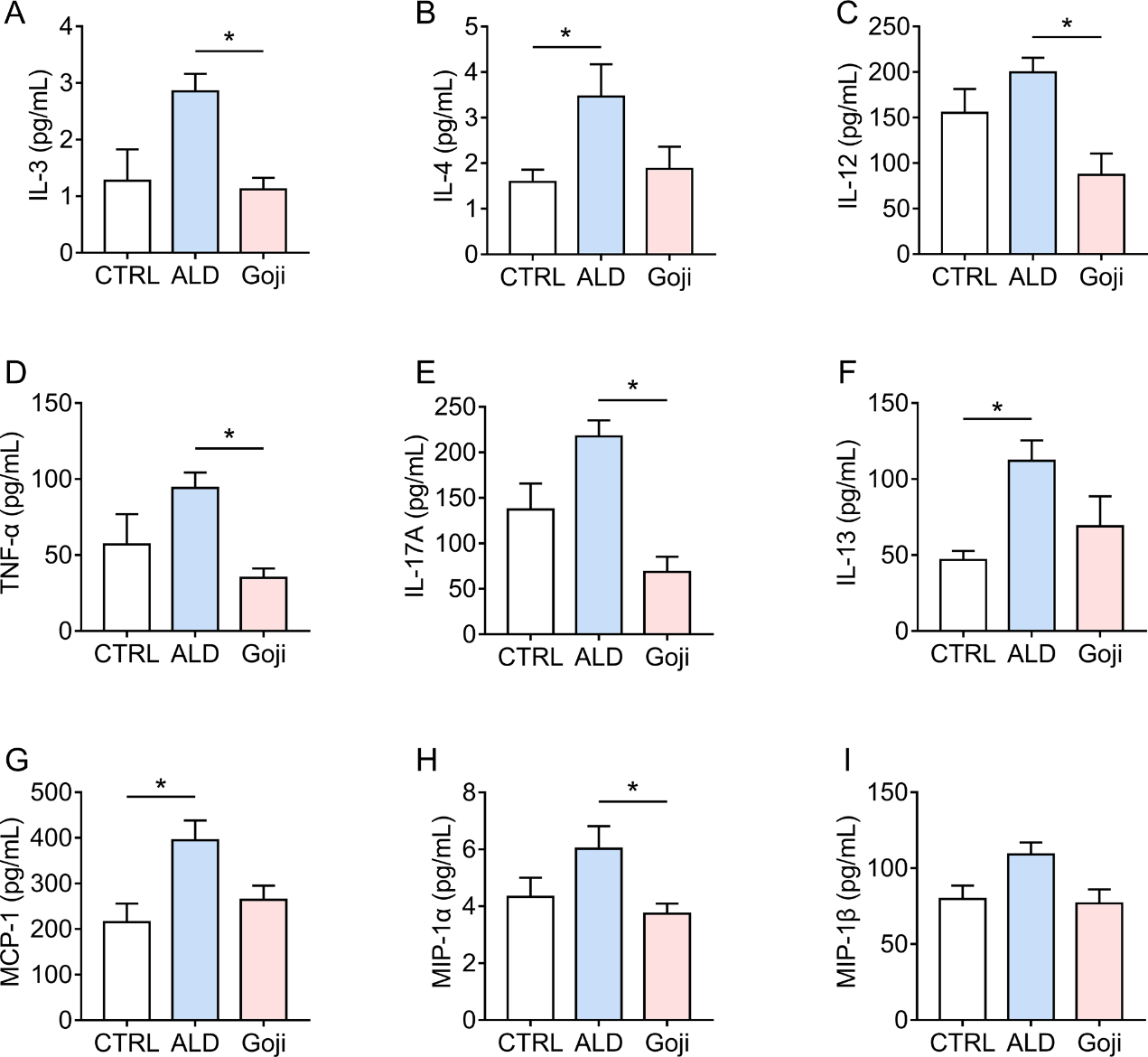
**

**Supplementary Figure 2.** The content of cytokines and chemokines in serum. **(A)** The contents of IL-3; **(B)** IL-4; **(C)** IL-12; **(D)** TNF-α; **(E)** IL-17A; **(F)** IL-13; **(G)** MCP-1; **(H)** MIP-1α; **(I)** MIP-1β. IL-3, interleukin-3; IL-4, interleukin-4; IL-12, interleukin-12; TNF-α, tumor necrosis factor alpha; IL-17A, interleukin-17A; IL-13, interleukin-13; MCP-1, monocyte chemotactic protein 1; MIP-1a, macrophage inflammatory protein-1 alpha; MIP-1β, macrophage inflammatory protein 1β. Data are shown as mean ± SEM (n=3-5), and statistical significance assessed by one-way ANOVA corrected for multiple comparison by Tukey test. **p*-value < 0.05.

**
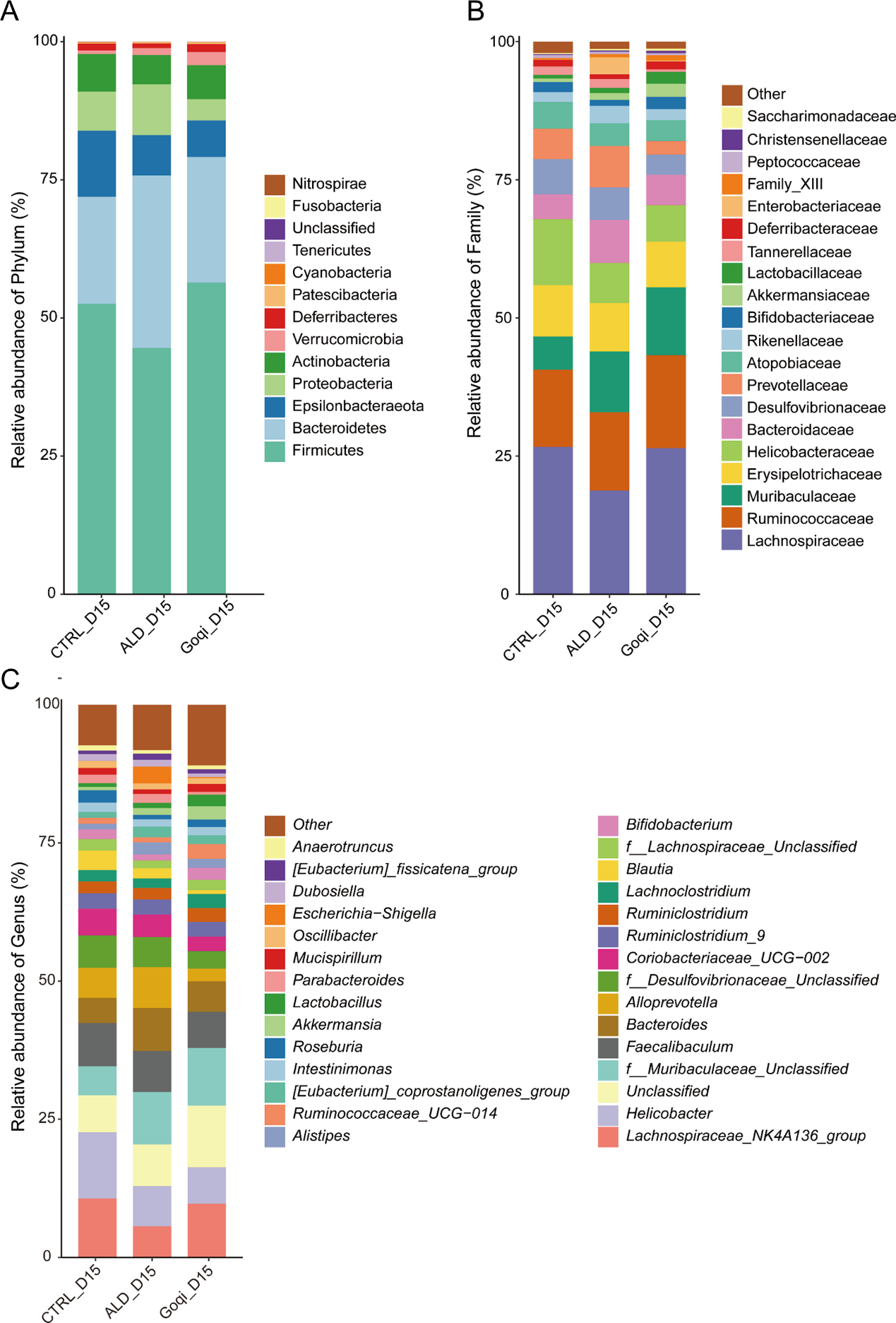
**

**Supplementary Figure 3.** The structure of gut microbiota in mice at day 15 (D15). **(A)** The relative abundance of gut microbiota at the phylum level; **(B)** The relative abundance of gut microbiota at the family level; **(C)** The relative abundance of gut microbiota at the genus level. Data are shown as mean ± SEM (n=7), and statistical significance assessed by one-way ANOVA corrected for multiple comparison by Tukey test. **p*-value < 0.05.

**
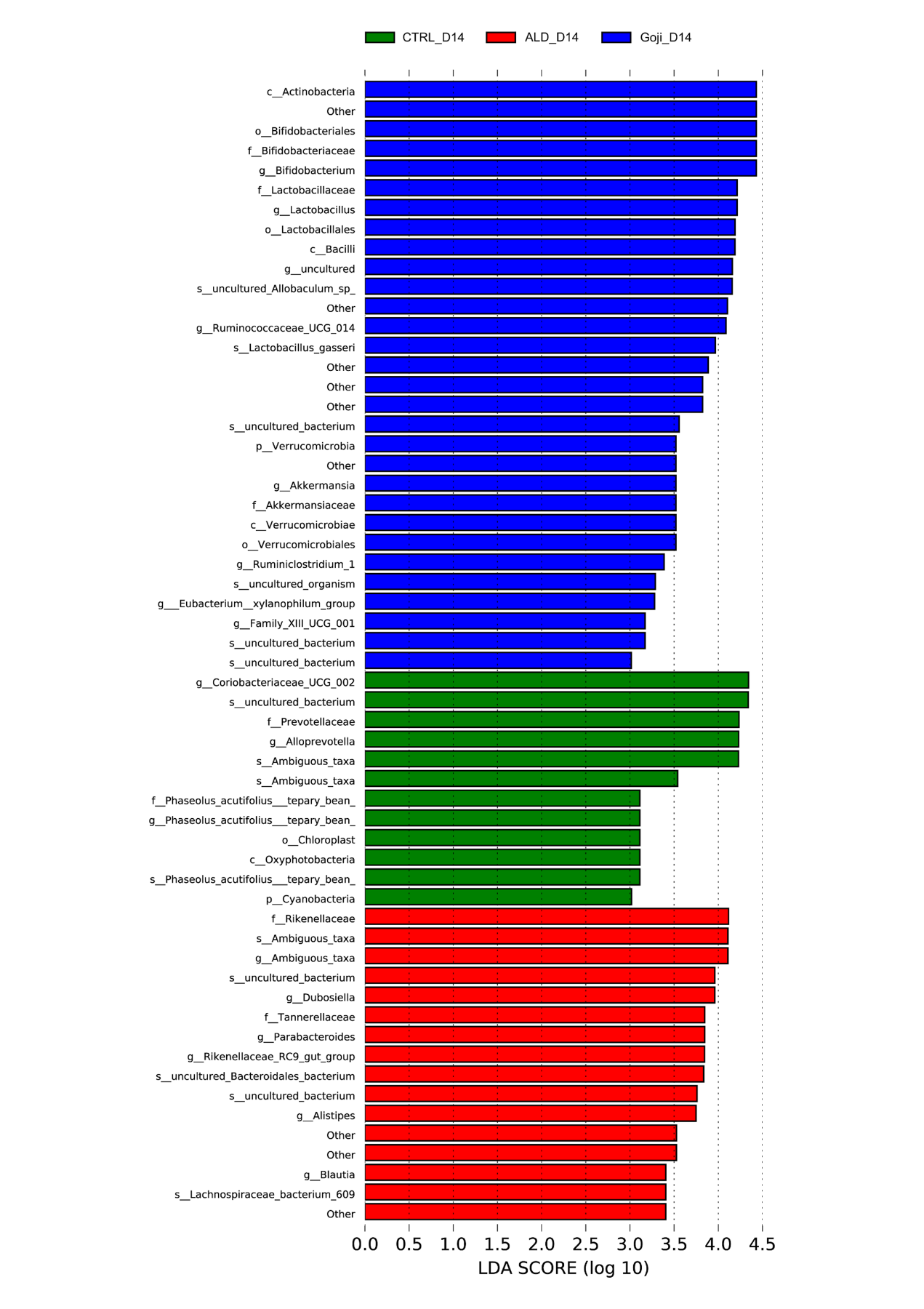
**

**Supplementary Figure 4.** Taxa enriched in CTRL (green), ALD (red) and Goji (blue) at day 14 (D14) are indicated with LDA scores. Only taxa meeting an LDA significant threshold of 3 are shown.

**
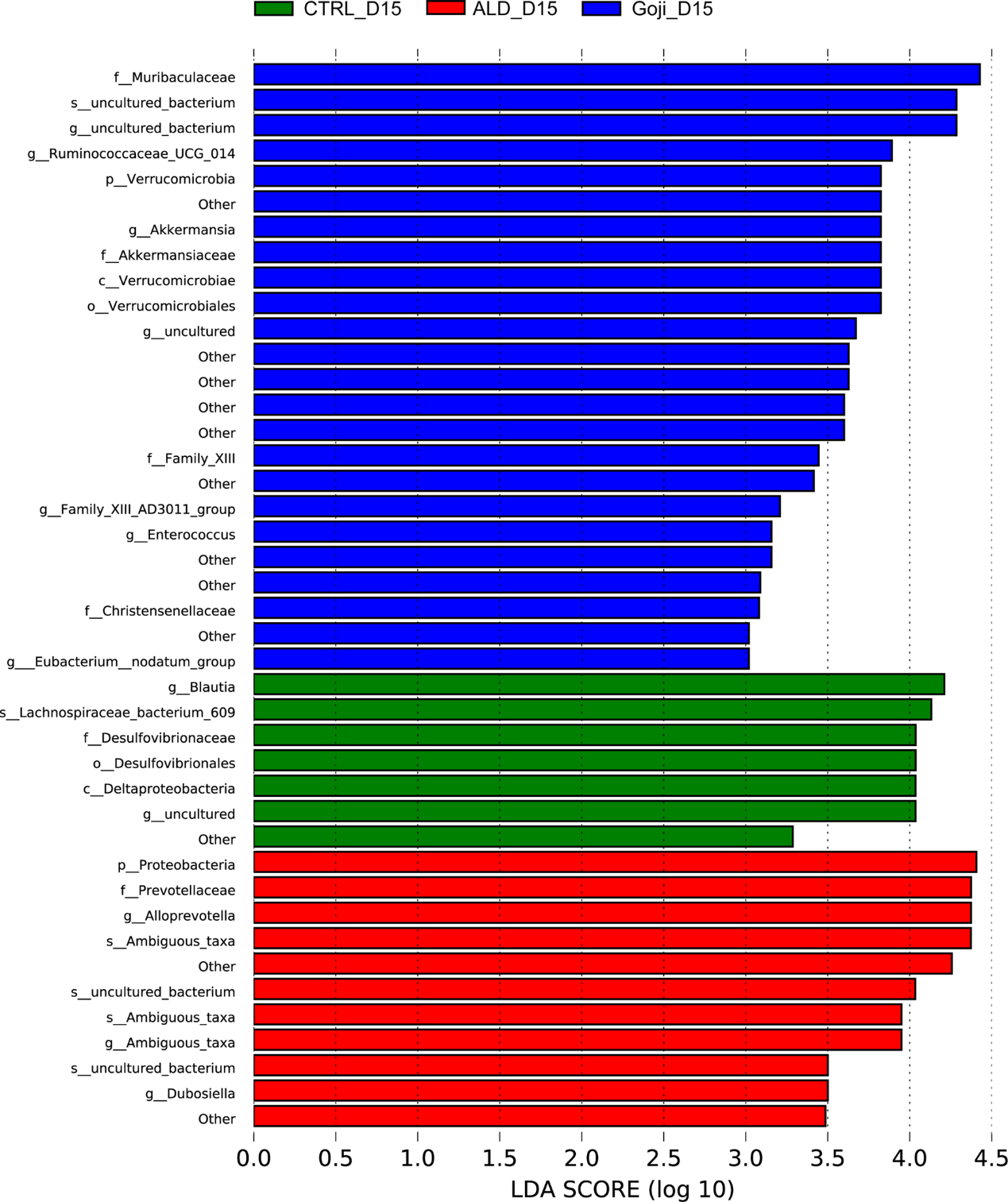
**

**Supplementary Figure 5.** Taxa enriched in CTRL (green), ALD (red) and Goji (blue) at day 15 (D15) are indicated with LDA scores. Only taxa meeting an LDA significant threshold of 3 are shown.

**
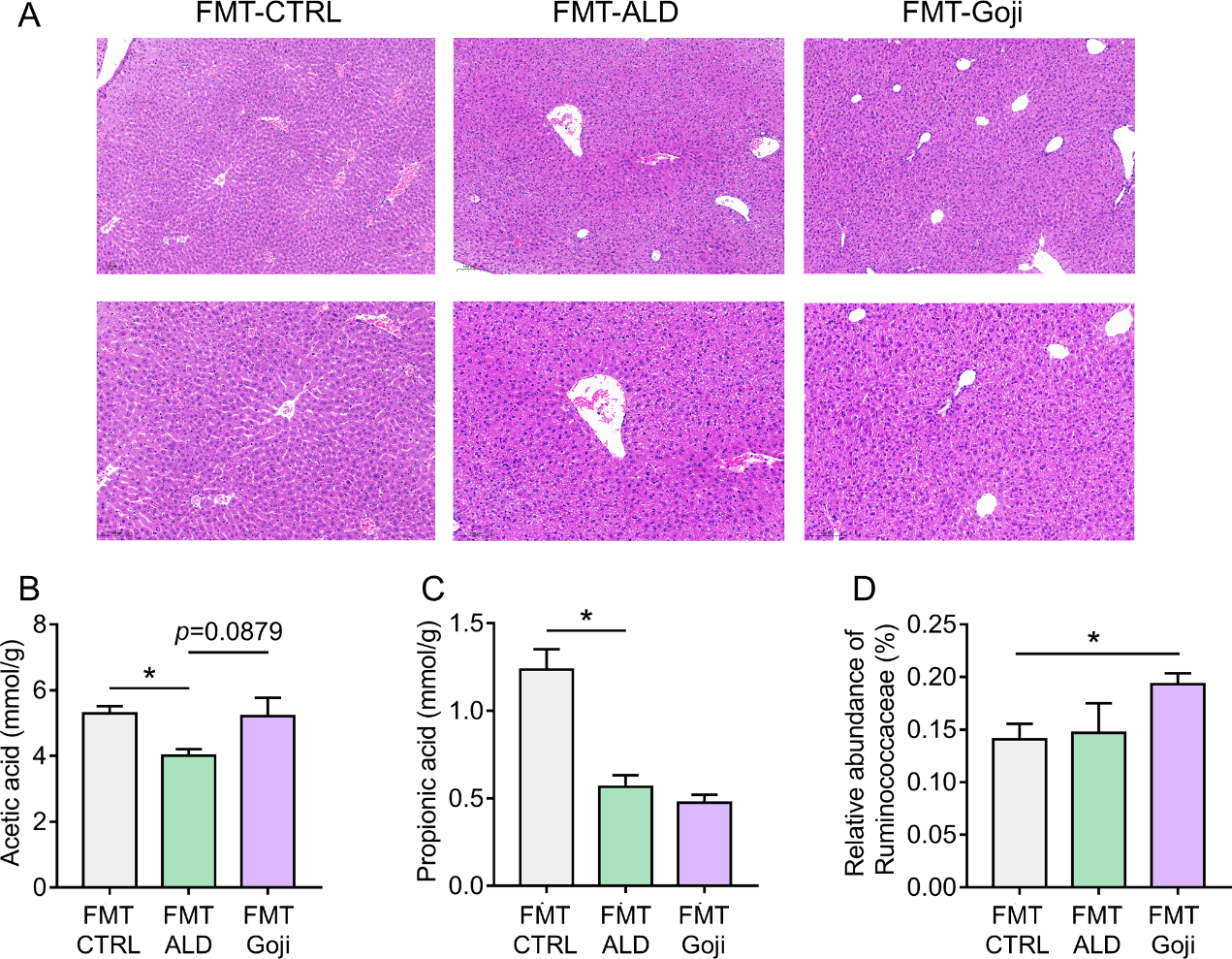
**

**Supplementary Figure 6.** **(A)** The representative sections of liver stained with hematoxylin and eosin; **(B)** Quantification of cecal acetic acid; **(C)** Quantification of propionic acid; **(D)** Relative abundance of family Ruminococcaceae.

# References

1. Cock PJ, Fields CJ, Goto N, Heuer ML, Rice PM. The Sanger FASTQ file format for sequences with quality scores, and the Solexa/Illumina FASTQ variants. *Nucleic Acids Res*. (2010) 38(6):1767-71. doi: 10.1093/nar/gkp1137

2. Langmead B, Salzberg SL. Fast gapped-read alignment with Bowtie 2. *Nat Methods*. (2012) 9(4):357-9. doi: 10.1038/nmeth.1923

3. Li B, Dewey CN. RSEM: accurate transcript quantification from RNA-Seq data with or without a reference genome. *BMC Bioinformatics*. (2011) 12:323. doi: 10.1186/1471-2105-12-323

4. Love MI, Huber W, Anders S. Moderated estimation of fold change and dispersion for RNA-seq data with DESeq2. *Genome Biol*. (2014) 15(12):550. doi: 10.1186/s13059-014-0550-8

5. Edgar RC. Search and clustering orders of magnitude faster than BLAST. *Bioinformatics*. (2010) 26(19):2460-1. doi: 10.1093/bioinformatics/btq461

6. Quast C, Pruesse E, Yilmaz P, Gerken J, Schweer T, Yarza P, et al. The SILVA ribosomal RNA gene database project: improved data processing and web-based tools. *Nucleic Acids Res*. (2012) 41(D1):D590-D6. doi: 10.1093/nar/gks1219
